# Validating the Utility of the Wilson Sex Fantasy Questionnaire With Men Who Have Sexually Offended Against Children

**DOI:** 10.3389/fpsyt.2019.00206

**Published:** 2019-04-09

**Authors:** Ross M. Bartels, Robert J. B. Lehmann, David Thornton

**Affiliations:** ^1^School of Psychology, University of Lincoln, Lincoln, United Kingdom; ^2^Department of Psychology, Medical School Berlin, Berlin, Germany; ^3^Sand Ridge Secure Treatment Center, Mauston, WI, United States

**Keywords:** sex offenders, sexual fantasy, Wilson Sex Fantasy Questionnaire, validity, crime scene behavior

## Abstract

The Wilson Sex Fantasy Questionnaire (WSFQ) assesses the use of 40 specific sexual fantasies, which are grouped into four overarching themes (Intimate, Exploratory, Impersonal, and Sadomasochistic). It also includes two items that reflect characteristics associated with children. Since sexual fantasies are a key factor in sex offender treatment, the present study tested the validity of the WSFQ for use with men who have sexually offended against children (SOC). Differential validity was assessed by comparing 54 SOC, 22 community males with a sexual interest in children (C-SI), and 79 community males with no sexual interest in children (C-NSI) on each WSFQ subscale and child-related item. Results showed that SOCs scored lower on each subscale than both community groups. On the two child-related items, the SOCs and C-SIs scored higher than C-NSIs. For the “Sex with someone much younger than yourself” item, younger SOCs had greater scores than younger C-NSIs, while older C-NSIs had greater scores than older SOCs. Construct validity was assessed using the SOC sample by examining relationships between WSFQ variables and 1) the self-reported use of deviant sexual fantasies assessed *via* the Thoughts and Fantasies Questionnaire and 2) offending behavior derived from crime scene data. The WSFQ Intimacy subscale was unrelated to any deviant sexual fantasies, while the other subscales were most strongly associated with sadistic fantasies. The child-related WSFQ items were most strongly associated with sexual fantasies about prepubescent children. Very few relationships were observed between the WSFQ variables and crime scene behaviors. The implications of the results are discussed, along with the study’s limitations and suggestions for future research.

## Introduction

Sexual fantasizing refers to the deliberate act of mentally envisioning a sexual scenario involving a target (e.g., a person) and/or behavior (e.g., dominating) (1). The content of the mental imagery generally reflects one’s sexual interest (2) and is experienced as sexually arousing (3). For example, in individuals who have sexually offended against a child, sexual fantasies involving children are often associated with a sexual interest in children (4) and are often used as a means of inducing or enhancing a state of sexual arousal (5).

Although sexual fantasizing is implicated in the etiology of child sexual abuse (6), a detailed understanding of how it actually influences offending behavior has yet to be established. Bartels and Gannon (7) highlight two ways, however, in which it may occur. The first refers to heightening an individual’s risk or propensity to sexually offend. That is, for those with a sexual interest in children, sexual fantasizing may psychologically and physiologically energize an individual (i.e., increase their sense of “wanting”), thus preparing them for engagement in sexually appetitive behavior (8, 9). When combined with masturbation and orgasm, this sense of wanting may be relieved in the short term but heightened in the medium term. The second link to offending is based on the idea that sexual fantasizing can create behavioral scripts (e.g., explicit or implicit plans) that an individual may enact in real life (5, 10, 11). Again, the inclusion of masturbation (and subsequent orgasm) is likely to strengthen the sexual meaning of the script, potentially increasing the likelihood of enacting the imagery in real life. Regardless of the exact causal mechanism, researchers have found that sexual fantasies about children are associated with contact sex offending behavior against children (4, 12, 13). While causality cannot be inferred from these findings given their correlational nature, sexual fantasizing is arguably an important factor to consider in the assessment and treatment of individuals who have sexually offended against children (SOCs). Thus, it is important for clinicians and researchers to have a reliable and valid tool for assessing sexual fantasy use.

One of the oldest and often used measures is the Wilson Sex Fantasy Questionnaire or WSFQ (14). The WSFQ includes a list of 40 sexual fantasy themes ranging from “the normal and innocuous to the deviant and relatively obscene” (15, p. 61). Each item is scored on a six-point scale ranging from Never (0) to Regularly (5), across five different contexts (i.e., Daytime fantasies, Fantasies during intercourse or masturbation, Dream while asleep, Have done in reality, and Would do in reality). When assessing the frequency of sexual fantasy use, Wilson (15) advises only using responses for Daytime fantasies, since scores for the other four contexts all highly correlate with Daytime fantasies. The WSFQ is composed of four factor analytically derived themes, each containing 10 items, namely, Intimate, Exploratory, Impersonal, and Sadomasochistic (14). This factor structure has been supported in subsequent confirmatory analyses, particularly in men (16). The WSFQ also provides a total score, which is argued to be a measure of one’s overall sex drive (15).

Only a few published studies have used the WSFQ with SOCs^1^. In one of the first studies, Baumgartner et al. (19) found that the WSFQ had very good internal consistency as indicated by Cronbach’s α (Intimate = .92; Exploratory = .86; Impersonal = .83; Sadomasochistic = .86; total score = .95). Below, we outline the studies that provide information about various forms of validity for the WSFQ.

Baumgartner et al. (19) also found that SOCs (*n* = 64) scored higher than nonsexual offenders (*n* = 41) on the Intimate and Exploratory subscales (*d* = 0.57 and 0.44, respectively). Crucially, they argued that two WSFQ items reflect themes associated with children (i.e., “Having sex someone much younger than yourself” and “Seducing an innocent”) and found that SOCs scored significantly higher than nonsexual offenders on these two items (*d* = 0.77 and 0.55, respectively). Baumgartner et al. (19) also compared their data to those reported in previous studies using college males (*N* = 116) (20), as well as non-offending fetishists (*N* = 24), sadomasochists (*N* = 34), and men with numerous sexual interests (*N* = 14) (15). The SOCs did not differ from college males on any subscales and reported lower Exploratory, Impersonal, and Sadomasochistic scores than the sadomasochistic and sexually variant males. However, they were unable to compare differences on the two child-related items.

Using a sample of 95 SOCs, Gannon et al. (21) examined differences between SOC subtypes (established by cluster analyzing data from a battery of measures). They identified five clusters, which they termed “Impulsive,” “Boy predators,” “Intimacy deficits,” “Generally antisocial,” and “Multiple dysfunction.” Discounting the “Multiple dysfunction” group due to a very small sample size (*n* = 4), it was found that, in contrast to the other groups, the “Boy predators” reported significantly higher scores on all WSFQ subscales, indicating higher levels of sexual fantasizing in general.

Other researchers have examined the WSFQ in relation to sexual recidivism in SOCs. Using an exploratory factor analysis with a sample of 495 SOCs, Allan et al. (22) found that the WSFQ subscales (pre-treatment) loaded on to a single factor. They labeled this factor “Sexual Interests,” stating that it “measures the strength of an offender’s sexual interest in terms of the frequency of their sexual fantasies” (p. 357). This factor, however, essentially represents the total WSFQ score and so does not provide any insight into the participants’ specific sexual interests. Nevertheless, this factor was found to be associated with sexual recidivism (Area Under the Curve; AUC = 0.72), suggesting that SOC’s frequency of fantasizing across an array of themes is predictive of sexual recidivism. A similar result was found by Stevens et al. (23) using a sample of 218 SOCs. Here, the same “Sexual Interests” factor (using WSFQ data) correlated with sexual recidivism, even after controlling for socially desirable responding (*r*_pb_ = .24). In addition, Stevens et al. (23) found that each WSFQ subscale was significantly associated with sexual recidivism (*r*_pb_ for Intimate = .15, Exploratory = .24, Impersonal = .19, Sadomasochistic = .18). Using the same dataset, Beggs and Grace (24) also found that positive change scores (following treatment) on the Sadomasochistic subscale were associated with reduced sexual recidivism (*r* = −.22).

Only a few studies have provided convergence data in terms of correlating the WSFQ with other indicators of deviant sexual interest. Using a sample of 302 sex offenders (type/s not specified), Seifert et al. (25) observed that the WSFQ total score strongly correlated with the sexual sensation-seeking and sexual compulsivity (*r* = .68 and .61, respectively). The WSFQ total also correlated strongly with the total score from a 90-item version of O’Donohue and Letourneau’s (26) Paraphilic Fantasy Questionnaire (*r* = .73) (27). Given that only the WSFQ total was used, a greater frequency of fantasizing across a range of themes is associated with sexual preoccupation (i.e., sexual compulsivity and sensation-seeking) and paraphilic sexual fantasies in general.

The link between fantasizing about sexual behaviors (e.g., sadomasochistic sexual fantasy themes) and objectively assessed offending behavior (e.g., sexualized aggression) in terms of construct validation has yet to be firmly established. This is rather surprising given the oft-described importance of sexual fantasies in forensic practice (28). This lack of research may be due to the range of issues pertaining to the study of sexual fantasies. For one, sexual fantasizing is a covert activity and is not externally identifiable outside of self-report. Moreover, from the point of view of someone who has offended, there may be little reward for being truthful about the content and use of one’s sexual fantasies in a forensic setting. They may even anticipate negative consequences for doing so (e.g., longer sentences, postsentence restrictions and requirements, stigma, physical violence threats from other inmates). As such, offenders are likely to have an understandable tendency for dissimulation. Accordingly, clinical subjective self-report data (e.g., WSFQ) may be of limited value in forensic assessments as they are easy to fake and may be biased by distorted self-perception and/or introspective abilities. This dissimulation hypothesis may be particularly true for sexual offenders whose behavior is (or was) driven by paraphilic interest compared to sexual offenders without an atypical sexual interest (i.e., those who offended because of a lack of more preferred sexual opportunities or general antisociality) (29).

As indicated above, there has been little validation of the WSFQ for use with SOCs. Most studies have primarily focused on the WSFQ subscales, have not accounted for sexual interest in children within comparison groups, and only examined its relationship with sexual offending behavior in terms of sexual recidivism. Thus, the aim of the present study was to further test the validity of the WSFQ for use with SOCs, taking into account the above issues. This goal was approached in three ways.

The first was to examine differential validity by comparing a sample of male SOCs with a sample of community males on the WSFQ subscales and child-related items. Recent findings indicate that some men from the general community report using sexual fantasies about children (30, 31), particularly those with a proclivity to engage in child sexual abuse (13). Therefore, we compared the SOCs with two subgroups of community men, namely, those reporting a sexual interest/proclivity for child sexual abuse, and those reporting no such interests. Based on Baumgartner et al. (19), it was hypothesized that SOCs would report using Intimate, Exploratory, and child-related sexual fantasies to a greater extent than community males with no sexual interest in children. We also predicted that SOCs would not differ from those with a sexual interest in children.

Second, we examined construct validity by correlating the WSFQ (i.e., its subscales and the two child-related items) with child-related and sadistic fantasies measured *via* another questionnaire designed to assess the use of offense-related sexual fantasies. It was hypothesized that, in terms of convergent validity, the Sadomasochistic subscale would positively correlate with sexual fantasies related to sexual sadism, while the child-related WSFQ items would correlate positively with sexual fantasies overtly involving children.

Third, construct validity was tested again. This was done by examining whether the WSFQ subscales and child-related items correlated with four behavioral themes identified in SOCs by Lehmann et al. (32) using crime scene data. These behavioral themes include a) Fixation (characterized by a persistent attraction to children), b) Regression [characterized by nonparaphilic sexual excitation and victim availability (e.g., in family setting) in response to intimacy deficits], (c) Criminality (where sexual abuse occurs in the context of generalized criminal behavior), and (d) Sexualized Aggression (characterized by offenses that involve overtly expressive aggression including behavioral indicators of sexual sadism). Accordingly, we hypothesized a positive relationship between the nonsexually deviant behavioral themes of Regression and Criminality and the normative and innocuous Intimate subscale. On the basis of the dissimulation hypothesis, we expected negative relationships between deviant behavioral themes (Fixation, Sexualized Aggression) and the WSFQ data.

## Method

### Sample

The offender sample was composed of 54 male individuals who had sexually offended against a child (i.e., aged 13 and younger), recruited from a secure treatment facility in the state of Wisconsin in the USA. The ages ranged from 25 to 73 years (*M* = 46.9, *SD* = 10.2). The majority (87%) had only sexually offended against a child, with the remaining 13% having sexually offended against both a child and an adult. Fifty-two (96.30%) reported being “single,” with one participant reporting being in a relationship, and another not providing his relationship status. Of the 50 participants with available information, the majority (77.8%, *n* = 42) had undergone or were undergoing some form of psychological treatment for their offending behavior at the time of data collection.

The non-offending sample was composed of 101 community males, who were all recruited online. The age of the community sample ranged from 18 to 51 years (*M* = 25.01, *SD* = 6.80), with 12 preferring not to provide their age. Fifty-seven of the non-offending sample (56.4%) reported being a relationship, while 44 (43.6%) reported being single.

### Data

Sexual fantasy data for the SOC sample were initially collected as part of a larger, distinct project led by one of the first authors. This initial project was aimed at exploring new indirect measures of sexual interest in children and offense-supportive cognition (33). Offense-related data were also available in some of the participants’ case files (*n* = 37). This allowed crime scene behaviors to be coded in the current study (see below for details). Sexual fantasy data for the community sample were drawn from a distinct online project examining child-related sexual interests in community males (Henek and Bartels, in preparation). Data for this initial project were collected online (using Qualtrics) *via* various social media platforms and forums (e.g., Twitter, Reddit). Each participant completed a small battery of measures assessing sexual compulsivity, sexual functioning, sexual fantasies (using the WSFQ), and sexual interest in children. In the current study, only the WSFQ data were used (for the group difference analyses). The data regarding sexual interest in children were used to categorize the community males into two groups: those reporting no sexual interest in children and those reporting some sexual interest in children (see below).

### Study Variables

#### Wilson Sex Fantasy Questionnaire (WSFQ) (14)

The WSFQ assesses how often people use 40 specific sexual fantasies. Each item is scored using a six-point scale (0 = Never, 5 = Regularly). The WSFQ is composed of four 10-item subscales: Exploratory (e.g., *Sex with two other people*), Intimate (e.g., *Having intercourse with a loved partner*), Impersonal (e.g., *Watching others having sex*), and Sadomasochistic (e.g., *Whipping or spanking someone*). Using the sample as a whole in the present study (*N* = 155), the WSFQ subscales showed acceptable to good levels of internal consistency: Impersonal (α = .68), Exploratory (α = .74), Sadomasochistic (α = .85), and Intimate (α = .87). The total score showed excellent internal consistency (α = .92).

Two specific sexual fantasy items were also of particular interest in this study. These were “Having sex with someone much younger than yourself” and “Seducing an innocent.” While these two items do not directly refer to children (e.g., a 50-year-old who fantasizes about a 25-year-old movie star may rate high on the former item), it has been argued that they involve “partners whose qualities could be seen as matching those of children (innocence and aged significantly younger)” (19, p. 28). We were additionally interested in the “Having incestuous sexual relations” item as it could reflect the offending behavior predominantly engaged in by SOC with a “regression” propensity (32). Please note that this item is rather vague and does not directly refer to children as well. Nonetheless, it was included given the exploratory nature of this part of the paper.

#### Thoughts and Fantasies Questionnaire

This is an unpublished questionnaire created by the third author for use in practice. It is designed to assess clients’ use of five specific deviant sexual fantasies during their time in treatment, namely, Abduction, Forcing, Children under 13 years, Children between 13 and 17 years old, and Sexual sadism. In addition, sexual fantasies involving the client’s past victim/s are also assessed. For each theme, a respondent first states whether they have experienced the sexual fantasy using a Yes/No format. If they respond with a “Yes,” they are required to answer a further set of open-ended questions (e.g., how often the fantasy was used, when it was last used, and how long it lasted). It also asks the respondent to write out the fantasy. In the present study, data from this measure were only available from the SOC sample.

#### The Interest in Child Molestation Scale (ICM) (34)

The ICM is a vignette-based self-report measure designed to assess community participants’ interest in sexual activity with children. The ICM is composed of five vignettes, each describing a hypothetical scenario of sexual activity with a child (age not specified). After reading each vignette, participants are required to report their level of sexual arousal, behavioral propensity (i.e., whether they would do the same), and general enjoyment. Each item is rated on a seven-point Likert scale (e.g., 1 = Not at all sexually aroused, 7 = Very strongly sexually aroused). Three of the vignettes involve low force and two involve high force. The ICM produces an overall score, a low-force subscale score, and a high-force subscale score. Previous studies using the ICM indicate that the low-force subscale is a particularly reliable and valid measure for assessing sexual interest in children in community samples (34, 35). On this basis, only the low-force subscale was administered to the community males in Henek and Bartels’ study (Henek and Bartels, in preparation). As there are nine items on the low-force subscale (rated on a Likert scale ranging from 1 to 7), the lowest possible score participants can obtain is 9 (i.e., no self-reported proclivity), with the highest possible score being 63.

In a study exploring pupillary responses as a method for assessing sexual interest, the ICM was used in a way to ensure that participants were solely interested in adults (36). Similarly, in the present study, the low-force subscale of the ICM (α = .82) was used to identify community males with a sexual interest in children (i.e., a score greater than 9). Of the 101 participants, 22 were identified as having some sexual interest in children (*M* = 14.55, *SD* = 5.60). These individuals were categorized as a “sexual interest in children” community group (C-SI), with the remaining 79 categorized as a “no sexual interest in children” community group (C-NSI).

### Coding

The offense-related information present in the offender participants’ case files was independently coded by two research assistants. This involved coding for the presence of 39 crime scene behaviors using the coding scheme devised by Lehmann et al. (32). Of the 54 available case files, 37 provided specific details that could be sufficiently coded. To determine inter-rater reliability for each variable, Cohen’s κ was computed. For seven variables (i.e., victim masturbates, offender offers money, offender films/photos victim, longer offense, ritualistic behavior, offender humiliates victim, and offender drugged victim), κ could not be computed due to a lack of variance. These variables, however, had high percent agreement (range = 95%–100%). For seven variables (affection, fondle, offender makes promises, luring, offender makes sexual comment, searching, and offender not deterred), κ coefficients were low (<.45) (37). Nevertheless, the variables aforementioned were included on the basis of the high percent agreement (range = 82%–92%) and because they were needed to compute propensity scores in order to test the link between the WSFQ and crime scene behavior (see **Supplementary Material** for full details). The κ coefficients for the remaining variables ranged from .52 to 1.00 (median = .76). After these initial codings, the first author examined each case file independently in order to provide the final decision on whether the crime scene behavior was present or not. Finally, following Lehmann et al. (32), the “present” crime scene variables associated with each behavioral theme (i.e., Fixation, Regression, Criminality, and Aggression) were averaged. This resulted in a continuous Thematic Sum Score (TSS) for the four behavioral themes.

### Analyses

Differences between the SOC, C-SI, and C-NSI groups on each WSFQ variable were assessed using one-way MANOVAs. Also, since the SOC group were (on average) older than the two comparison groups, we examined whether any group effect on the two child-related WSFQ items were moderated by participant age. Next, in the SOC sample only, relationships between WSFQ variables and the child-related and sadistic themes of the Thoughts and Fantasies Questionnaire were examined using rank-biserial correlations (controlling for age). Finally, for those SOCs with available crime scene data (*n* = 37), Spearman’s Rho correlations (controlling for age) were run to explore whether scores on the WSFQ were associated with the TSS scores derived from crime scene data. Given the multiple correlations (i.e., 38), a Bonferroni correction was employed to adjust for the familywise error rate (α changed from .05 to .0013).

## Results

### Differential Validity

A one-way, independent-samples MANOVA was used to compare the three groups (SOC vs. C-SI vs. C-NSI) on the four WSFQ subscales (i.e., Intimate, Exploratory, Impersonal, and Sadomasochistic). A significant multivariate main effect of Group was observed [Wilks’ λ = 0.69, *F*(8, 298) = 7.48, *p* < .001, η_p_^2^ = 0.17]. As shown in **Table 1**, there was a significant main effect of Group for each WSFQ variable, except for Exploratory (*p* = .09). This lack of difference on the Exploratory subscale was in line with our hypothesis with respect to SOCs and C-SIs, but not SOCs and C-NSIs. *Post hoc* comparisons indicated that C-NSIs used Intimate fantasies significantly more so than SOCs (*p* = .001, *d* = 0.62). While this is counter to our hypothesis derived from Baumgartner et al.’s (19) findings, it is understandable that community males with no interest in children would report higher scores on this normative subscale. The lack of a difference between SOCs and C-SIs was, however, as expected. For Impersonal fantasies, C-SI had significantly greater scores than both the SOC (*p* = .001, *d* = 0.92) and C-NSI (*p* = .03, *d* = 0.65). The C-SIs also reported using Sadomasochistic fantasies more often than SOCs (*p* < .001, *d* = 1.17), as did the C-NSIs (*p* < .001, *d* = 1.08).

**Table 1:** Descriptive and inferential statistics for group differences on each Wilson Sex Fantasy Questionnaire (WSFQ) scale.

WSFQ subscale	SOC (*n* = 54)	C-SI (*n* = 22)	C-NSI (*n* = 79)			
	*M* (*SD*)	*M* (*SD*)	*M* (*SD*)	*F*	*p*	*η*_p_^2^
Intimate	25.31^a^ (11.82)	30.91^ab^ (8.29)	31.85^b^ (9.23)	6.99	.001	.08
Exploratory	13.85^a^ (9.02)	18.55^a^ (8.79)	15.11^a^ (7.86)	2.43	.09	.03
Impersonal	11.96^a^ (7.93)	19.00^b^ (7.43)	14.38^a^ (6.86)	19.54	<.001	.21
Sadomasochistic	4.65^a^ (6.21)	14.64^b^ (10.38)	12.76^b^ (8.67)	7.22	.001	.09

SOC, sexual offenders against children; C-SI, community males with a sexual interest in children; C-NSI, community males with no sexual interest in children.

Groups that share superscripts (i.e., a or b) do not significantly differ (p < .05, Bonferroni corrected).

A second independent-samples MANOVA was conducted to examine group differences for the two specific WSFQ items of interest (i.e., “Having sex with someone much younger,” “Seducing an innocent”). There was a significant multivariate main effect of Group [Wilks’ λ = 0.88, *F*(4, 302) = 3.50, *p* = .001, η_p_^2^ = 0.06]. A significant main effect was observed for both items (*p’*s < .01; see **Table 2**). *Post hoc* comparisons revealed that SOCs scored significantly higher than C-NSIs on “Sex with someone much younger” (*p* = .001, *d* = 0.67), as did C-SIs (*p* = .001, *d* = 0.82). For “Seducing an innocent,” SOCs reported marginally greater and non-negligible scores (based on effect size) than C-NSIs (*p* = .056, *d* = 0.44), while C-SIs reported significantly greater scores than C-NSIs (*p* = .02, *d* = 0.62). These findings were in line with our hypotheses.

**Table 2:** Descriptive and inferential statistics for group differences on child-related WSFQ items.

WSFQ item	SOC (*n* = 54)	C-SI (*n* = 22)	C-NSI (*n* = 79)			
	*M* (*SD*)	*M* (*SD*)	*M* (*SD*)	*F*	*p*	*η*_p_^2^
Someone much younger	1.96^a^ (1.64)	2.32^a^ (1.91)	0.92^b^ (1.47)	10.32	<.001	.12
Seducing an innocent	1.48^a^ *_m_* (1.56)	1.86^a^ (1.91)	0.87^b^ *_m_* (1.20)	5.31	.006	.07

SOC, sexual offenders against children; C-SI, community males with a sexual interest in children; C-NSI, community males with no sexual interest in children.

Groups that share superscripts (i.e., a or b) do not significantly differ (p < .05, Bonferroni corrected). Groups that share an m subscript are marginally different.

**Figure 1:**
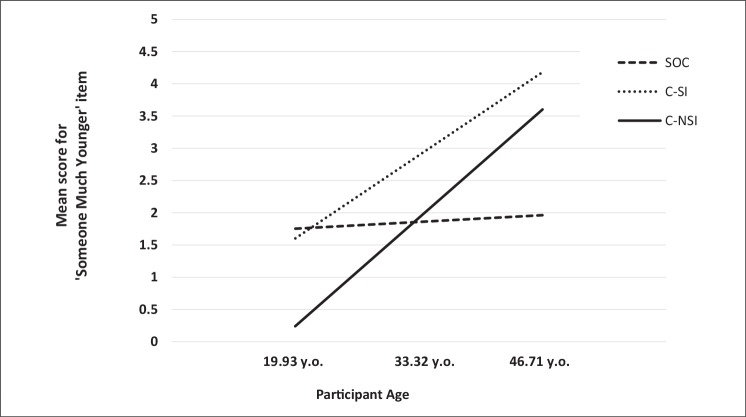
“Someone much younger than yourself” scores as a function of group and participant age.

**Figure 2:**
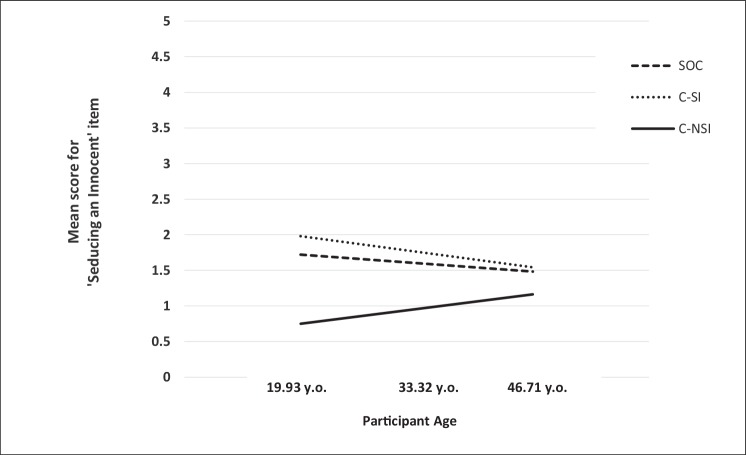
“Seducing an innocent” scores as a function of group and participant age.

Given that the “child-related” WSFQ items do not actually explicitly refer to children (but rather youth and innocence), it is possible that the older participants interpreted them innocuously (e.g., in terms of a much younger *adult*). This could account for why SOCs scored high on these items, as they were significantly older than C-NSIs and C-SIs (both *p*’s < .001). Thus, to examine whether participant age had a moderating effect on the link between group and the item “Sex with someone much younger,” we used the PROCESS macro for SPSS (38). As there were three groups, the independent variable was specified as being multicategorical using indicator coding (39), with SOCs coded as the reference group. Age was found to have a significant moderating effect [Δ*R* ^2^ = 0.07, *F*(2, 137) = 6.83, *p* = .002]. However, this was only in relation to the difference between SOCs and C-NSIs (*b* = .12, *SE* = .03, *t* = 3.60, *p* < .001), not between SOCs and C-SIs (*b* = .09, *SE* = .06, *t* = 1.62, *p* = .11). As shown in **Figure 1**, the conditional effects revealed that, at lower age levels (−1 *SD* below the mean), scores on the “Someone much younger” item were greater for SOCs than for C-NSIs (*b* = 1.51, *SE* = 0.61, *t* = 2.47, *p* = .02). There was no difference between the two groups at medium (mean) age levels (*b* = .06, *SE* = 0.44, *t* = 0.14, *p* = .87), but at higher age levels (+1 *SD* above the mean), C-NSIs showed greater scores on the item (*b* = 1.64, *SE* = 0.62, *t* = 2.64, *p* = .009). Conversely, the relationship between group and “Seducing an innocent” was found to not be moderated by participant age, Δ*R* ^2^ = 0.005, *F*(2, 137) = 0.34, *p* = .71 (see **Figure 2**).

### Construct Validity: Correlations Between Sexual Fantasy Measures

Data from both sexual fantasy measures were available for all SOCs. Rank-biserial correlations (controlling for age) between the WSFQ variables and the dichotomous responses on the Thoughts and Fantasies Questionnaire (TFQ) themes are presented in **Table 3**. As shown, only four correlations survived the Bonferroni correction for multiple testing. In line with our hypotheses, the Sadomasochistic subscale showed a positive relationship with the Sadistic theme of the TFQ (*r*_rb_ = .46) (convergent validity), as did the Impersonal subscale to a stronger degree (*r*_rb_ = .51). As expected, the Intimate subscale did not correlate with any of the examined TFQ themes (discriminant validity). Also as hypothesized, the “Sex with someone much younger” and “Seducing an innocent” WSFQ items both showed moderate-to-strong, positive correlations with the Child <13 TFQ theme (*r*_rb_ = .43 and .48, respectively). No significant relationships were found between the single WSFQ items and the postpubescent (Child 13–17 years) TFQ theme.

**Table 3:** Rank-biserial correlations between sexual fantasy measures within the sexual offenders against children (SOC) sample, controlling for age.

	Thoughts and Fantasies Questionnaire Themes		
	Child (< 13 years)	Child (13–17 years)	Sadistic acts
**WSFQ variables**			
Intimate WSFQ	.07	.23	.20
Exploratory WSFQ	.29	.14	.38**
Impersonal WSFQ	.30*	.04	.46***
Sadomasochistic WSFQ	.10	.20	.51***
“Sex with someone much younger”	.43***	.23	.32*
“Seducing an innocent”	.48***	.24	.33*

*p < .05, **p < .01, ***p ≤.001. SOC, sex offenders against children.

Results in **bold** typeface indicate significant results after Bonferroni correction (α = .0013).

### Construct Validity: Relationship Between Sexual Fantasies and Behavioral Themes

Crime scene data and sexual fantasy data were available for 37 SOCs. **Table 4** shows Spearman’s correlations (controlling for age) between the WSFQ variables and the four behavioral themes derived from crime scene information (i.e., TSS scores). None of the observed relationships were significant after applying the Bonferroni correction. Thus, we did not find support for the hypothesis that the nondeviant themes (Regression and Criminality) would correlate with the Intimate subscale. However, from looking at the size of the correlations, two relationships are worth noting (both of which were significant before corrections). First, the Intimate subscale showed a moderate negative relationship with the “Sexualized Aggression” TSS (*r*_rho_ = −.34) and, in line with expectations, the Regression theme showed a moderate relationship with “Incestuous sexual relations” (*r*_rho_ = .33). Also, in line with the dissimulation hypothesis, both behavioral themes indicating sexual deviance (Fixation and Sexualized Aggression) consistently showed negative relations with the WSFQ data (except between Fixation and the Sadomasochistic subscale).

**Table 4:** Spearman correlations between the WSFQ variables and Thematic Sum Scores, controlling for age.

WSFQ variables	Fixation	Regression (sexualization)	Criminality	(Sexualized) aggression
Intimate subscale	−.08	−.08	−.03	−.34*
Sadomasochistic subscale	.14	.04	.26	−.14
“Sex with someone much younger”	−.03	−.11	−.01	−.18
“Seducing an innocent”	−.23	−.09	−.21	−.12
“Incestuous sexual relations”	−.16	.33*	−.18	−.22

*p < .05. WSFQ = Wilson Sex Fantasy Questionnaire. Results in **bold** typeface indicate significant results after Bonferroni correction (α = .0013).

## Discussion

The present study investigated the utility of the Wilson Sex Fantasy Questionnaire (WSFQ) in relation to its use with individuals who have sexually offended against children (SOC). Previous research using the WSFQ with SOC samples have 1) focused primarily on the broad subscales and total score (which provide no information about child-related sexual fantasy themes), 2) failed to account for sexual interest in children within the comparison group/s, and 3) focused on sexual recidivism, rather than specific offending behaviors. The present study aimed to take into account these three points.

Counter to our hypotheses, which were based on the findings of Baumgartner et al. (19), SOCs did not score higher than community males with no sexual interest in children (C-NSIs) on the Intimate and Exploratory subscales. Rather, C-NSIs scored higher than SOCs on the Intimate subscale. As predicted, SOCs and community males with a self-reported sexual interest in children (C-SIs) did not differ on these two subscales. In addition, SOC’s scores on the Sadomasochistic subscale were much lower than that reported by C-SIs and C-NSIs, while C-SIs reported using Impersonal sexual fantasies more frequently than both the SOCs and C-NSIs. These findings could suggest some level of dissimulation of sexual fantasies in SOCs compared to community males. However, SOCs have been found to score lower than sadomasochistic and sexually variant men on the Sadomasochistic subscale, as well as the Exploratory and Impersonal subscales (19). Thus, the community males in the present study may have been particularly sadomasochistic. Indeed, our C-NSI group had much higher scores on the Sadomasochistic subscale than the college males in Plaud and Bigwood’s (20) study (*M* = 12.76 vs. 4.9, respectively). Thus, it is possible that the present study used a biased (self-selected) community sample (i.e., one composed of sadistic and/or sexually variant individuals). However, as social desirability was not accounted for in this study, the dissimulation hypothesis cannot be discounted.

As hypothesized, the SOCs scored higher on the two child-related WSFQ items (“Seducing an innocent” and “Having sex with someone much younger than yourself”) than the C-NSI group, as did the C-SIs. These results provide partial support for Baumgartner et al.’s (19) proposition that these two WSFQ items assess fantasy content related to children.

It should be noted, however, that the difference between SOCs and C-NSIs for “Sex with someone much younger” was moderated by participant age. This reflected a relatively stable level of fantasy use in the SOCs, but an increase in use for the C-NSIs. Thus, at a younger age, SOCs scored higher than C-NSIs, while at an older age, C-NSIs scored higher than SOCs. A similar trend was also observed for C-SIs (see **Figure 1**). This highlights an issue with the ambiguous terminology for this particular item (40). That is, the phrase “someone much younger” can mean different things for younger and older individuals. For younger men, it may be more likely to be interpreted as “children,” which could account for why the SOCs (and C-SIs) scored greater than C-NSIs at a younger age. For older men, though, the item may be more likely be interpreted as a much younger adult.

In terms of convergence, correlational analyses (controlling for age) indicated that, within the SOC sample, the two child-related WSFQ items were most strongly associated with sexual fantasies about children under 13 years old (as measured by the Thoughts and Fantasies Questionnaire; TFQ). These findings provide further validation that these two items may tap a sexual interest in child-related characteristics. Other notable correlations were in relation to the Sadomasochistic and Impersonal subscales, both of which showed strong links with sexual fantasies about sadistic acts on the TFQ.

After correcting for multiple correlations, none of the relationships (controlling for age) were significant in relation to the behavioral themes derived from crime scene data. However, focusing on the size of the correlations, the Intimate subscale showed a moderate, negative association with the Sexualized Aggression theme (potentially attesting to discriminant validity). Also, the “Incestuous sexual relations” item showed a moderate positive correlation with the Regression theme. This aligns with Lehmann et al.’s (32) findings showing that the Regression theme was related to the closeness of the victim–offender relationship, indicating proximity to incestuous relations. However, despite the size of the correlations, it should be emphasized that they did not survive the conservative Bonferroni correction we applied. A clear pattern of relationships was observed, however, that suggested some level of dissimulation in the SOCs. That is, the deviant sexual fantasy TFQ themes were, overall, negatively related to deviant behavioral themes (albeit nonsignificantly). Thus, subjective self-report data may be of less value when assessing individuals in forensic contexts. Also, it is important to keep in mind that the SOC’s sexual fantasies were assessed many years after their initial offense had been committed, as well as after undergoing treatment for their deviant sexual fantasies and related factors.

Nevertheless, taken together, the current findings offer some implications for research and practice. First, “Seducing an innocent” and “Sex with someone much younger than yourself” from the WSFQ both substantially correlated with sexual fantasies about young children in SOCs and distinguished sexually deviant community males (and SOCs at younger ages) from those with no sexual interest in children. Joyal et al. (40) argued that these two items are ambiguous and so, on their adapted WSFQ, they removed “Seducing an innocent” and amended the latter to “Sex with someone much younger (legally) than me.” However, our findings suggest that these two items may reflect characteristics associated with children (i.e., “youth” and “innocence”), as suggested by Baumgartner et al. (19). It could be argued that individuals with a sexual interest in children may find these characteristics particularly appealing and, thus, incorporate them into their sexual fantasies (with fantasies involving children being the extreme manifestation of these characteristics). This is analogous to “dominance”—a characteristic associated (but not synonymous) with rape—that is sexually fantasized about by men who have sexually aggressed (41, 42). Taken together, researchers and clinicians may be able to use the “Seducing an innocent” and “Sex with someone much younger” WSFQ items as proxies for assessing child-related sexual fantasies, or as a means to identify the potential use of such fantasies. Arguably, this may be a more favorable approach, as items that overtly ask about fantasies involving children are likely to provoke faked responses. As our data suggest, however, it should be kept in mind that the ambiguity of the “Sex with someone much younger” item introduces issues for older respondents.

Second, this study highlights an important consideration for future comparative studies on the topic of SOC’s sexual fantasies. That is, a sexual interest in children should be taken into account when collecting data from comparison groups. This will allow researchers to either screen out those with an interest in children (providing a purer comparison group) or form two comparison groups based on the presence or absence of a sexual interest in children (as in this study). Failing to account for a sexual interest in children within comparison groups will likely lead to biased interpretations (e.g., about the target and/or comparison group, or the measure that is being tested).

### Limitations and Future Research

In addition to potentially having recruited a biased online sample of community males, further limitations should be noted. First, other comparison groups could have been included, such as a group of nonsexual offenders or a sexual offender comparison group (e.g., rapists). If these groups were found to score lower on the child-related items, it would provide further validation of the WSFQ for use with SOC populations. Second, the majority of the SOC sample had received treatment for their offending behavior. Since treatment has been shown to significantly reduce scores on the Exploratory, Impersonal, and Sadomasochistic WSFQ subscales (43), it is possible that similar reductions had also occurred for many of the SOCs in this study. In spite of this possibility, the child-related items still correlated with the prepubescent child theme on the TFQ in SOCs. Third, most SOCs self-reported as being “single,” whereas many of the non-offender participants were in relationships. This may have affected the use of normative sexual fantasies, potentially accounting for why SOCs did not score higher than C-NSIs on the Intimate subscale. Fourth, it is important to note that social desirable responding was not accounted for. Thus, given the possible indication of dissimulation in this study, future research should include impression management measures to control for response biases.

Finally, the exploratory findings regarding the relationship between sexual fantasies and offending behavior must be interpreted with some caution. First, due to a lack of sufficient crime scene information, only data from a much smaller sample could be coded. A larger sample with more detailed crime scene information would have been more desirable. Second, the sexual fantasy data in this study were collected (often long) after the offending behavior had occurred. Therefore, it is possible that the SOCs had been fantasizing about behaviors and targets unrelated to their prior offending behavior (either due to treatment-related or age-related changes). Future research should consider using a sample of recently convicted SOCs or those at a pre-treatment stage.

### Conclusion

The results of this study suggest that using the WSFQ with SOCs may be more useful than just assessing broad fantasy themes (*via* subscales). That is, two items (“Seducing an innocent” and “Sex with someone much younger”) contain characteristics associated with children. As such, they may be useful proxies for assessing child-related sexual fantasies for occasions when asking directly about children is problematic or particularly sensitive. However, given their vague terminology, issues with interpretation of these items (especially in older individuals) is something that should be carefully considered. The Sadomasochistic subscale also appears to be a valid means for assessing sadistic interests within SOCs. Further work is still needed regarding convergent validity (e.g., with other measures of sexual interest), predictive validity in relation to sexual recidivism, and differential validation (e.g., between SOC subtypes).

## Ethics Statement

This specific study was carried out in accordance with the recommendations of the “British Psychological Society” guidelines and was approved by the School of Psychology Research Ethics Committee at the University of Lincoln. All participants tested in person provided written informed consent, while those who took part online provided informed consent by clicking the relevant response (i.e., “I Agree”).

## Author Contributions

RB formulated the research question and designed the study, analyzed the data, and cowrote the article. RL formulated the research question and designed the study, helped with interpretation of the findings, and cowrote the article. DT provided important intellectual content and proofread the article.

## Conflict of Interest Statement

The authors declare that the research was conducted in the absence of any commercial or financial relationships that could be construed as a potential conflict of interest.

## Footnotes

Some researchers have adapted the WSFQ by adding, removing, and/or amending items (17, 18). For the purposes of this paper, however, only studies using the original WSFQ are discussed.

## References

[1] Bartels RM Beech AR Theories of deviant sexual fantasy. In: Boer DP Beech AR Ward T The Wiley handbook on the theories, assessment, & treatment of sexual offending. Volume I: Theories, Chichester, UK: John Wiley & Sons (2016). 10.1002/9781118574003.wattso008

[2] Chivers ML A brief review and discussion of sex differences in the specificity of sexual arousal. Sex Relation Ther (2005) 20:377–90. 10.1080/14681990500238802

[3] Leitenberg H Henning K Sexual fantasy. Psychol Bull (1995) 117:469–96. 10.1037/0033-2909.117.3.469 7777650

[4] Stinson JD Becker JV Assessing sexual deviance: a comparison of physiological, historical, and self-report measures. J Psychiatr Pract (2008) 14:379–88. 10.1097/01.pra.0000341892.51124.85 19057239

[5] Gee DG Ward T Eccleston L The function of sexual fantasies for sexual offenders: a preliminary model. Behav Change (2003) 20:44–60. 10.1375/bech.20.1.44.24846

[6] Ward T Siegert RJ Toward a comprehensive theory of child sexual abuse: a theory knitting perspective. Psychol Crime Law (2002) 8:319–51. 10.1080/10683160208401823

[7] Bartels RM Gannon TA Understanding the sexual fantasies of sex offenders and their correlates. Aggress Violent Behav (2011) 16:551–61. 10.1016/j.avb.2011.08.002

[8] Seto MC The Motivation-facilitation model of sexual offending. Sex Abuse (2019) 31:3–24. 10.1177/1079063217720919 28715948

[9] Smid WJ Wever EC Incentive theory of sexual motivation: a framework for the description of sexual offending behaviour and the role of sexual deviance. In: Boer DP Beech AR Ward T The Wiley handbook on the theories, assessment, & treatment of sexual offending. Volume I: Theories, Chichester, UK: John Wiley & Sons (2016). 10.1002/9781118574003.wattso007

[10] Pithers WD Relapse prevention with sexual aggressors: a method for maintaining therapeutic gain and enhancing external supervision. In: Marshall WL Laws DR Barbaree HE, editors. Handbook of sexual assault: Issues theories and treatment of the offender. New York: Plenum Press (1990). p. 343–61. 10.1007/978-1-4899-0915-2_20

[11] Ward T Hudson SM Sexual offenders’ implicit planning: a conceptual model. Sex Abuse (2000) 12:189–202. 10.1023/A:1009534109157 10904991

[12] Klein V Schmidt AF Turner D Briken P Are sex drive and hypersexuality associated with pedophilic interest and child sexual abuse in a male community sample? PLoS One (2015) 10. 10.1371/journal.pone.0129730 PMC4492978 26147099

[13] Turner-Moore T Waterman M Men presenting with sexual thoughts of children or coercion: flights of fancy or plans for crime? J Sex Med (2017) 14:113–24. 10.1016/j.jsxm.2016.11.003 27915076

[14] Wilson GD The secrets of sexual fantasy. London: J.M. Dent & Sons (1978).

[15] Wilson G Measurement of sex fantasy. Sex Marital Ther (1988) 3:45–55. 10.1080/02674658808407692

[16] Sierra JC Ortega V Zubeidat I Confirmatory factor analysis of a Spanish version of the Sex Fantasy Questionnaire: assessing gender differences. J Sex Marital Ther (2006) 32:137–59. 10.1080/00926230500442318 16418105

[17] Cortoni F Marshall WL Sex as a coping strategy and its relationship to juvenile sexual history and intimacy in sexual offenders. Sex Abuse (2001) 13:27–43. 10.1023/A:1009562312658

[18] Renaud CA Byers ES Positive and negative sexual cognitions: subjective experience and relationships to sexual adjustment. J Sex Res (2001) 38:252–62. 10.1080/00224490109552094

[19] Baumgartner JV Scalora MJ Huss MT Assessment of the Wilson Sex Fantasy Questionnaire among child molesters and nonsexual forensic offenders. Sex Abuse (2002) 14:19–30. 10.1023/A:1013025410090 11803593

[20] Plaud JJ Bigwood SJ A multivariate analysis of the sexual fantasy themes of college men. J Sex Marital Ther (1997) 23:221–30. 10.1080/00926239708403927 9292837

[21] Gannon T Terriere R Leader T Ward and Siegert’s Pathways Model of child sexual offending: a cluster analysis evaluation. Psychol Crime Law (2012) 18:129–53. 10.1080/10683160903535917

[22] Allan M Grace RC Rutherford B Hudson SM Psychometric assessment of dynamic risk factors for child molesters. Sex Abuse (2007) 19:347–67. 10.1177/107906320701900402 17874186

[23] Stevens CD Tan L Grace RC Socially desirable responding and psychometric assessment of dynamic risk in sexual offenders against children. Psychol Crime Law (2016) 22:420–34. 10.1080/1068316X.2015.1120868

[24] Beggs S Grace RC Treatment gain for sexual offenders against children predicts reduced recidivism: a comparative validity. J Consult Clin Psychol (2011) 79:182–92. .org/10.1037/a0022900 10.1037/a0022900 21341890

[25] Seifert K Boulas J Huss MT Scalora MJ Response bias on self-report measures of sexual fantasies among sexual offenders. Int J Offender Ther Comp Criminol (2017) 61:269–81. 10.1177/0306624X15593748 26160536

[26] O’Donohue W Letourneau EJ Dowling H Development and preliminary validation of a paraphilic sexual fantasy questionnaire. Sex Abuse (1997) 9:167–78. 10.1007/BF02675062

[27] Skovran LC Huss MT Scalora MJ Sexual fantasies and sensation seeking among psychopathic sexual offenders. Psychol Crime Law (2010) 16:617–29. 10.1080/10683160902998025

[28] Vanhoeck K Van Daele E Gykiere K Fantasy management in sex offender treatment. Sex Offender Treat (2011) 6 www.sexual-offender-treatment.org/94.html.

[29] Seto MC Pedophilia and sexual offending against children: theory, assessment, and intervention. Washington, DC: American Psychological Association (2008). 10.1037/11639.000

[30] Dombert B Schmidt AF Banse R Briken P Hoyer J Neutze J How common is men’s self-reported sexual interest in prepubescent children? J Sex Res (2016) 52:214–23. 10.1080/00224499.2015.1020108 26241201

[31] Santtila P Antfolk J Räfså A Hartwig M Sariola H Sandnabba NK Men’s sexual interest in children: one-year incidence and correlates in a population-based sample of Finnish male twins. J Child Sex Abuse (2015) 24:115–34. 10.1080/10538712.2015.997410 25747416

[32] Lehmann RJ Goodwill AM Hanson RK Dahle KP Crime scene behaviors indicate risk-relevant propensities of child molesters. Crim Justice Behav (2014) 41:1008–28. 10.1177/0093854814521807

[33] Bartels RM Beech AR Harkins L Thornton D Assessing sexual interest in children using the Go/No-Go Association Test. Sex Abuse (2018) 30:593–614. 10.1177/1079063216686119 28100118

[34] Gannon TA O’Connor A The development of the Interest in Child Molestation Scale. Sex Abuse (2011) 23:474–93. 10.1177/1079063211412390 22031298

[35] Mitchell RC Galupo MP Interest in child molestation among a community sample of men sexually attracted to children. J Sex Aggress (2015) 22:224–32. 10.1080/13552600.2015.1056263

[36] Attard-Johnson J Bindemann M, Ciardha CÓ. Pupillary response as an age-specific measure of sexual interest. Arch Sex Behav (2016) 45:855–70. 10.1007/s10508-015-0681-3 PMC4820473 26857377

[37] Altman DG Practical statistics for medical research. London: Chapman & Hall (1991).

[38] Hayes AF Introduction to mediation, moderation, and conditional process analysis: a regression-based approach. New York, NY: The Guilford Press (2013).

[39] Hayes AF Montoya AK A tutorial on testing, visualizing, and probing an interaction involving a multicategorical variable in linear regression analysis. Commun Methods Meas (2017) 11:1–30. 10.1080/19312458.2016.1271116

[40] Joyal CC Cossette A Lapierre V What exactly is an unusual sexual fantasy? J Sex Med (2015) 12:328–40. 10.1111/jsm.12734 25359122

[41] Moyano N Sierra JC Sexual victimisation, sexual cognitions, desire and excitation/inhibition in community Spanish male and female sexual aggressors. J Sex Aggress (2016) 22:36–51. 10.1080/13552600.2014.996614

[42] Renaud CA Byers ES Relationship between sexual violence and positive and negative cognitions of sexual dominance. Sex Roles (2005) 53:253–60. 10.1007/s11199-005-5683-5

[43] Beggs SM Grace RC Assessment of dynamic risk factors: an independent validation study of the Violence Risk Scale: Sexual Offender Version. Sex Abuse (2010) 22:234–51. 10.1177/1079063210369014 20458126

