# Supplementary material for: Validating the Utility of the Wilson Sex Fantasy Questionnaire With Men Who Have Sexually Offended Against Children
Source: Front Psychiatry. 2019 Apr 9;10:206. doi: 10.3389/fpsyt.2019.00206 (PMC6465618; doi:10.3389/fpsyt.2019.00206)
Supplement: Supplementary file 1 [file Table_1.docx]

**Supplementary material**

**Table:** Kappa and agreement indices for coding of crime scene variables (*N* = 38)

| **Crime Scene Behaviour** | **Kappa** | **Percent agreement** |
| --- | --- | --- |
| Male victim | .90 | 87% |
| Affection | .37 | 87% |
| Fondle | .44 | 90% |
| Stranger victim | .60 | 87% |
| Offender makes promises | .36 | 92% |
| Luring | .23 | 87% |
| Outdoors | .84 | 95% |
| Offender makes sexual comment | .36 | 82% |
| Searching | .09 | 84% |
| Kiss | .89 | 97% |
| Ejaculation | .85 | 95% |
| Victim masturbates offender | .72 | 95% |
| Offender performs oral | .52 | 84% |
| Offender masturbates | .53 | 92% |
| Fellatio | .64 | 84% |
| Money | N/A | 100% |
| Offender shows porn | 1.00 | 100% |
| Victim masturbates | N/A | 100% |
| One-off | .74 | 82% |
| Force control | .62 | 90% |
| Verbal violence | .79 | 97% |
| Safety precautions | .41 | 82% |
| Initial force | .77 | 95% |
| Threatens no report | .55 | 90% |
| Threatened to kill | 1.00 | 100% |
| Weapon | 1.00 | 100% |
| Offender intrudes | 1.00 | 100% |
| Anal | .83 | 95% |
| Film/photo | N/A | 100% |
| Injury to sexual organ | .66 | 97% |
| Longer offense (> 1 hr) | N/A | 97% |
| Force physical | .64 | 95% |
| Posing | 1.00 | 100% |
| Not deterred | .10 | 26% |
| Ritualistic behaviour | N/A | 97% |
| Penetration with object | .54 | 92% |
| Offender is intoxicated | .66 | 97% |
| Humiliate | N/A | 95% |
| Drugged | N/A | 100% |
